# Systematic Approach to the Synthesis of Cobalt-Containing Polyoxometalates for Their Application as Energy Storage Materials

**DOI:** 10.3390/ma16145054

**Published:** 2023-07-17

**Authors:** Ángela Barros, Beñat Artetxe, Unai Eletxigerra, Estibaliz Aranzabe, Juan M. Gutiérrez-Zorrilla

**Affiliations:** 1 Surface Chemistry and Nanotechnologies Unit, Tekniker, Iñaki Goenaga 5, 20600 Eibar, Spain; unai.eletxigerra@tekniker.es (U.E.); estibaliz.aranzabe@tekniker.es (E.A.); 2 Departamento de Química Orgánica e Inorgánica, Facultad de Ciencia y Tecnología, Universidad del País Vasco UPV/EHU, 48080 Bilbao, Spain; benat.artetxe@ehu.eus (B.A.); juanma.zorrilla@ehu.eus (J.M.G.-Z.)

**Keywords:** polyoxometalate, cobalt, synthesis, energy storage materials, electroactive species, electrolyte

## Abstract

New energy storage materials are an object of study within the framework of the global energy transition. The development of renewable sources is being boosted thanks to stationary energy storage systems such as redox flow batteries (RFBs). This work reports the synthesis of the cobalt-containing Keggin-type polyoxometalates [CoW_12_O_40_]^6−^ (**CoW_12_**) and [Co(H_2_O)SiW_11_O_39_]^6−^ (**CoSiW_11_**), which have previously been shown to have applicability in RFBs. These procedures were reassessed to meet the strict requirements associated with the further implementation of RFBs, including fast and affordable synthetic procedures with high reaction yields. In contrast to the lengthy and complicated synthetic approaches published to date, the optimized synthesis reported in this work enables the isolation of the pure crystalline salt of the **CoW_12_** anion with a 75% reduction of the time of the whole reaction procedure, eliminating tedious steps such as the recrystallization and including a 20% increased yield. The control of the stoichiometry, fine-tuning of reaction conditions, and the identification of intermediate species, as well as the acidic equilibria taking place during the process, were monitored via thermal, spectroscopic, and structural analyses. In the case of the **CoSiW_11_** anion, its preparation was based on a simple and highly efficient procedure. Moreover, promising electrochemical properties were observed with the use of the one-pot synthetic approach, in which the stoichiometric amounts of the starting reagents are dissolved in the supporting electrolyte to be directly implemented as the electrolyte for a RFB.

## 1. Introduction

Polyoxometalates (POMs) are a rich family of inorganic metal–oxygen clusters that constitute a unique class of compounds [1]. Due to their special reactivity together with their structural and compositional versatility, they are employed in different areas such as materials science [2], catalysis [3], and medicine [4]. The simplest procedures for the synthesis of POMs start from aqueous solutions of [MO_4_]^m−^ (M = V, Mo, W) oxyanions that undergo complex self-assembly processes via acidic condensation equilibria of {MO_6_} polyhedral subunits until the formation of the desired polyanion is achieved [5]. Afterwards, clusters are precipitated by the addition of a suitable counterion (e.g., alkali metals, organic cations). These complex formation equilibria are highly influenced by chemical and physical stimuli; hence, their fine-tuning is a key factor in the control of the formation of the desired POMs with high yield and purity [6]. Extensive research has been carried out over the years on the optimization of the synthetic procedures for the formation of POMs [7]. Consequently, long lists of variables that are crucial for the formation of given clusters have been identified. These include concentration and type of the metal source, ionic strength and pH, sequence of the addition of reagents, and external physical (e.g., temperature and pressure) and chemical factors (i.e., presence of counterions, heteroatoms, or ligands), among others.

Recently, POMs have been an object of study because of their potential as redox-active species for redox flow batteries (RFBs). RFBs are electrochemical energy storage systems that are currently a topic of interest due to the necessary transition from fossil fuels to renewable sources [8]. It has been proposed that they could supply the grid during periods of demand due to the instability of renewable energy sources. They present a unique architecture which enables easy and affordable scalability, as well as a fast response and long life. Their mechanism of energy storage consists of the direct exploitation of chemical reactions involving electron transfers [9], which enables the possible implementation of different chemistries [8,10]. As a result of this architecture, the energy density of RFBs is directly dependent on the concentration of the electroactive species on the electrolyte; thus, an important approach to extend the potential of this technology is to develop high-yield and fast methods to synthesize the redox-active species. 

In this regard, POMs display special electrochemical properties that make them ideal candidates as electroactive species for these electrolytes. Some POMs are considered electron reservoirs because they can accept several electrons without undergoing changes in their structure, due to the electron delocalization that takes place throughout all the metal centers. For example, a Keggin-type {XM_12_O_40_} POM could undergo up to 24 electron transferences [11]. Electron transfers are usually linked to protonation or association of cations, which maintains the net charge of POM species as a constant and provides them with increased stability. In addition, POMs usually undergo fast and reversible multi-electron transfers, which enables high energy densities to be achieved [12]. Moreover, the versatility of POMs allows the modification of their electrochemical properties with slight compositional and structural variations that can be achieved with the control of the synthetic procedures. This compositional versatility enables them to present two metal centers in the same polyanion; hence, different redox reactions can take place in the same molecule, which allows symmetric cell configurations to be built.

POM-based RFBs were first designed in 2013 by Pratt et al., based on the electrochemical activity of the vanadium-trisubstituted Keggin-type polyanion [HSiV_3_W_12_O_40_]^6−^ [13]. Following this study, many further works were reported. These included the use of vanadium-substituted Keggin- (e.g., A-α-[PV_3_W_9_O_40_]^6−^ and B-α-[PV_3_W_9_O_40_]^6−^), Wells–Dawson- (e.g., [P_2_V_3_W_15_O_62_]^9−^ [14]), and Lindqvist-type (e.g., cis-[V_2_W_4_O_19_]^4−^ [15]) polyoxotungstates, which showed promising performance results. Furthermore, organically functionalized POMs were also tested (e.g., [TiV_5_O_6_(OCH_3_)_13_]^−^ [16], [V_6_O_7_(OCH_3_)_12_] [17], and TBA_3_[PW_11_O_39_(SiC_6_H_5_)_2_O] [18]) in an attempt to increase the potential of the system by using organic solvents, but the limited reversibility of the redox processes restricted their electrochemical performance. Nevertheless, recently, aqueous POM-based electrolytes for use in high-operating-potential RFBs have been developed [19,20]. Interesting results were also obtained for the transition-metal-containing [CoW_12_O_40_]^6−^ species in a symmetric cell configuration [21], because Co (Co^II^W^VI^/Co^III^W^VI^) and W (Co^II^W^VI^/Co^II^W^V^) centers can be oxidized and reduced, respectively. The fact that cross-contamination was avoided because of the presence of identical species in both the anolyte and catholyte led to higher efficiencies than those displayed previously by asymmetric systems. In addition, the work by Friedl et al. is remarkable [22], as they firstly developed a scaled-up POM-based RFB set up which showed enhanced efficiency in comparison to those previously assessed at laboratory scale. Moreover, H_6_P_2_W_18_O_62_ has shown high power density as an aqueous negolyte even under extreme conditions (−20 °C) [23]. In general terms, the great variety of POM-based RFBs developed in recent years [24] exemplifies the potential of these systems. In addition, POMs are also receiving attention in other kinds of energy storage systems, for example as redox mediators in fuel cells [25,26], which enhances their potential in the field of energy transition. Nevertheless, there is plenty of room to increase their efficiency and boost this new technology to be competitive in the energy storage market.

One of the main drawbacks that hinders the further exploitation of POMs as energy storage materials are the time-consuming synthetic procedures with low yields, which significantly increase the total cost of production. Herein, we report on a systematic approach for the optimization of the synthesis of two cobalt(II)-containing Keggin-type anions that have been selected as potential electroactive species for a symmetric and aqueous RFB. The selected POMs are the plenary and monosubstituted species [α-CoW_12_O_40_]^6−^ (hereafter **CoW_12_**) and [α-Co(H_2_O)SiW_11_O_39_]^6−^ (hereafter **CoSiW_11_**), respectively. After the careful optimization of the electrolyte composition, they exhibited a suitable operational potential as well as high reversibility of the redox processes in an appropriate medium, namely 1M acetic acid/lithium acetate (H/LiAc) buffer solution [19]. Structurally, the well-known α-Keggin-type structure is constituted by four W_3_O_13_ trimers formed by three edge-sharing WO_6_ octahedra, which are linked to each other and to the central XO_4_ tetrahedron by corner-sharing. In **CoW_12_**, the cobalt(II) atom occupies the heteroatomic position X, whereas in **CoSiW_11_**, one of the shell W=O units is substituted by a Co(H_2_O) moiety. Different synthetic parameters, including starting reagents, initial stoichiometry, and the formation of secondary products, were rigorously examined. As a result, the preparation procedure has been improved in terms of purity, atomic economy, time efficiency, and reaction yield, revealing the feasibility of the development of POM-based electrolytes for the potential implementation of RFBs in the electric grid.

## 2. Materials and Methods

All the reagents were purchased from commercial sources (Scharlab, Barcelona, Spain) and used without further purification. Metal analyses were performed using a Quadrupole Inductively Coupled Plasma Mass Spectrometry (Q-ICP-MS) Thermo XSeries-II analyzer (Thermo Fisher Scientific, Waltham, MA, USA). Fourier-transform infrared (FT-IR) spectra were obtained using KBr pellets on a Shimadzu FTIR-84000S spectrometer (Shimadzu, Kyoto, Japan). Powder X-ray diffraction (PXRD) patterns were recorded using a Philips X’PERT PRO diffractometer (Philips, Eindhoven, The Netherlands) operating at 40 mV/40 mA in θ-θ configuration with 2θ = 5 to 50° (0.03° step size, 30 s per step) and monochromate Cu Kα radiation (λ = 1.5418 Å) with a PIXcel detector. Thermogravimetric analyses (TGA) were performed on a LF1 Mettler Toledo thermobalance (Mettler Toledo, Columbus, OH, USA) with a 50 cm^3^ min^−1^ flow of synthetic air, from room temperature to 800 °C at a rate of 5 °C min^−1^. Ultraviolet–visible (UV-Vis) spectra were recorded on a JENWAY 6300 spectrophotometer (Cole Parmer Inc., Saint Neots, UK) on a wavelength scan range of 400 to 800 nm. Semiquantitative analyses were performed using a Bruker S8 Tiger 4K wavelength dispersive X-ray fluorescence (WD-XRF) spectrometer (Bruker, Billerica, MA, USA) with a rhodium X-ray source at 50 kV. X-ray photoelectron spectroscopy (XPS) measurements were performed using a SPECS system (SPECS Surface Nano Analysis, Berlin, Germany) equipped with a Phoibos 150 1D-DLD analyzer (Berlin, Germany) and an Al Kα monochromatic radiation source (1486.6 eV). Electrochemical studies were performed using a BioLogic VSP-3e potentiostat with EC-Lab v11.36 software (Biologic Science Instruments, Seyssinet-Pariset, France). Cyclic voltammetry (CV) was recorded with a three-electrode cell (20 mL), using a polished glassy carbon electrode (GCE) (0.07 cm^2^) as the working electrode, Ag/AgCl (in 3M KCl) as the reference electrode, and a Pt wire counter electrode at a scan rate of 50 mV s^−1^. The conductivity of the electrolytes was measured using a Crison 5072 conductivity probe associated with a MM 41 Crison multimeter.

### 2.1. Synthesis of K_4_H_2_[CoW_12_O_40_]·12H_2_O (**K-CoW_12_**)

The **CoW_12_** anion was synthesized by modifying procedures reported in the literature [27]. To an aqueous solution (120 mL) of Na_2_WO_4_·2H_2_O (60 g, 180 mmol) acidified to pH 7.5 with glacial acetic acid, Co(CH_3_CO_2_)_2_·4H_2_O (7.47 g, 30 mmol) dissolved in water (52 mL) was added dropwise and the mixture was kept under reflux conditions. After 20 min, solid K(CH_3_CO_2_) (60 g) was added and the solution was stirred for an additional 10 min. Then, the resulting solid was filtered under vacuum and an emerald green solid K_8_[Co_2_(H_2_O)W_11_O_39_]·17H_2_O (**K-Co_2_W_11_**) was obtained. Yield: 96% based on W. UV-Vis (H_2_O) λ_max_ (nm): 600. FT-IR $\bar{\nu }$ (KBr, cm^−1^): 929 (s), 860 (s), 781 (s), 660 (m), 540 (w), 459 (m).

The **K-Co_2_W_11_** salt was dissolved in acidic medium with a 1 g:10 mL 1M HCl ratio. After five days, needle-like dark blue crystals of K_4_H_2_[CoW_12_O_40_]·12H_2_O were isolated after slow evaporation of the final solution at room temperature. Overall yield: 88% based on W. Elemental analyses: Co 1.77%, K 4.75%, W 67.29%. Calculated for CoH_26_K_4_O_52_W_12_: Co 1.82%, K 4.82%, W 67.96%. UV-Vis (H_2_O) λ_max_ (nm): 624. FT-IR $\bar{\nu }$ (KBr pellet, cm^−1^): 943 (s), 881 (s), 736 (s), 447 (s). Powder XRD: ICSD-89706 [28]; TGA (50 cm^3^ min^−1^ synthetic air; 5 °C min^−1^) dehydration process that extended from room temperature up to 250 °C (found 6.2%, 6.5% calculated for 12 H_2_O). Final residue calculated for CoK_4_W_12_O_39_ (found): 92.9% (93.1%). 

### 2.2. One-Pot Preparation of **CoSiW_11_** Electrolyte 

Na_2_WO_4_·2H_2_O (55 mmol, 18.15 g), Na_2_SiO_3_ (5 mmol, 0.6 g), and Co(CH_3_CO_2_)_2_·4H_2_O (5 mmol, 1.25 g) were consecutively added to a boiling aqueous 1M H/LiAc buffer solution (150 mL). The color of the solution changed from pale to dark red after 12 h, as monitored using UV-Vis spectroscopy, which confirmed the total formation of the [Co(H_2_O)SiW_11_O_39_]^6−^ (**CoSiW_11_**) polyanion. After filtering any solid out, the electrolyte was ready for the electrochemical tests. Yield: 78% determined by UV-Vis (1M H/LiAc), λ_max_ (nm): 546.

## 3. Results and Discussion

### 3.1. Optimization of the Synthetic Procedure for K_4_H_2_[CoW_12_O_40_] (**K-CoW_12_**)

The plenary anion [CoW_12_O_40_]^6−^ was first isolated in 1956 by Baker et al. [27]. In that work, the authors studied the reactivity of Co(II)-containing Keggin-type heteropolyoxotungstates towards acids and oxidizing agents. The proposed synthesis comprised an aqueous solution of the starting reagents (cobalt(II) acetate and sodium tungstate) in a 1:6 stoichiometry ratio which succeeded for a relatively wide range of pH values (6.5–7.5). After ten minutes of heating, a green intermediate solid was precipitated with ammonium acetate, which was later identified as an ammonium salt of the [Co_2_(H_2_O)W_11_O_39_]^8−^ (**Co_2_W_11_**) anion on the basis of elemental analyses. In order to obtain the desired [CoW_12_O_40_]^6−^ (**CoW_12_**), it was necessary to dissolve the intermediate in highly acidic conditions (1M HCl) and to let it evaporate at room temperature until dark blue needle-like crystals were formed. The product was obtained at a 70% yield, but four recrystallization processes were needed to obtain a pure crystalline product. Although a short reaction time can be advantageous, the whole procedure takes several weeks to complete because up to four recrystallizations are needed to obtain a pure compound. This synthetic procedure has been reproduced over the years with slight modifications [28,29,30], but the nature of the side products has never been studied to our knowledge.

**Figure 1:**
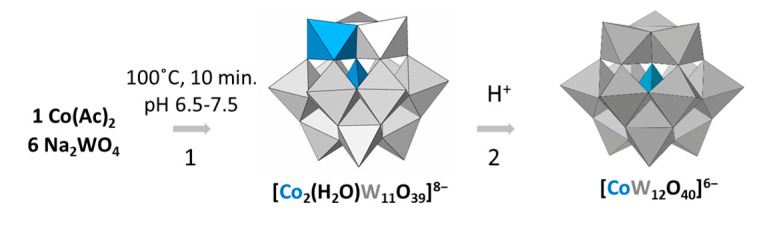
Scheme of the stepwise synthesis of **CoW_12_** via isolation of the intermediate **Co_2_W_11_** species (Reaction 1) and acidic condensation of the former to lead to the plenary anion (Reaction 2).

The reaction takes place in two main steps. First, the **Co_2_W_11_** anion is precipitated from a one-pot synthesis involving metal precursors at neutral pH conditions. Afterwards, the **Co_2_W_11_** salt is dissolved in a highly acidic medium to shift the equilibrium towards the plenary **CoW_12_** anion (Figure 1). These two Keggin-type POMs are structurally related, in that both display a central {CoO_4_} tetrahedron in a heteroatomic position. In contrast, unlike the plenary anion, the substituted **Co_2_W_11_** species exhibits an additional {Co(H_2_O)} moiety substituting one of the shell W=O subunits. Transformation from monosubstituted to plenary species upon acidification is one of the most representative reactions involving Keggin-type POMs, which increase their degree of condensation with the decreasing pH of the medium, whereas vacant lacunary species are formed upon basic degradation of plenary species [1].

In this scenario, we evaluated whether the reaction time and the total cost of the process could be considerably reduced while increasing the reaction yield in order to further assess the exploitation of POM-based electrolytes for RFBs. Therefore, a detailed investigation of the whole reaction procedure is reported herein. It was carried out using characterization techniques such as powder X-ray diffraction (PXRD), Fourier-transform infrared (FT-IR) spectroscopy and thermogravimetric analysis (TGA).

#### 3.1.1. Optimization of the pH Conditions for the Formation of **Co_2_W_11_**

In the original synthetic procedure, the pH is adjusted between 6.5 and 7.5 using acetic acid before the addition of the cobalt salt [27]. This is a wide pH range considering the complex pH dependency of the formation equilibria of POMs in water, because many species could be coexisting in solution [31]. Thus, the pH value of the reaction was finely tuned with glacial acetic acid (strong acids were dismissed due to the difficulties in controlling the pH) to 6.5, 7, 7.5, and 8. In the last step, precipitation of the anion was achieved by using potassium acetate. The results summarized in Table 1 reveal that the reaction yield for the formation of **Co_2_W_11_** showed a strong pH dependency. The highest reaction yield (96%) was obtained at pH 7.5, in which no side product was formed. Lower pH values led to pink precipitates that were identified as cobalt(II) salts of the [H_2_W_12_O_42_]^10−^ paratungstate-A anion on the basis of FT-IR spectroscopy (Figure S1) and semiquantitative analysis using XRF (Calculated for K_6_[{Co(H_2_O)_4_}_2_(H_2_W_12_O_42_)] Co 3.84%, W 72.93%; Anal. Co 3.85%, W 82.12%) [32]. This assumption is plausible when taking into account that the pH values are within the well-known stability range of paratungstate species [33]; hence, the reaction could easily take place as indicated in Equation (1). These solids formed in the reaction process needed to be filtered out before precipitating the **Co_2_W_11_** anion as a potassium salt.

In contrast, pH values above 7.5 afforded a dark oily product, the liquid nature of which made its further characterization difficult. In view of these results, we can conclude that the fine-tuning of the initial pH not only increased the reaction yield by almost 50% (10% more than the highest reported values in previous works), but also avoided an additional filtering step for the isolation of **K-Co_2_W_11_** as a pure crystalline phase.

12 WO_4_^2−^ + 2Co^2+^ + 14H^+^ + 2 H_2_O ↔ [Co_2_W_11_O_39_]^8−^ + WO_4_^2−^ + 4 H^+^ + 7 H_2_O ↔ [(Co(H_2_O)_4_)_2_(H_2_W_12_O_42_)]^6−^ (1)

#### 3.1.2. Stoichiometry

Although the Co:W atomic ratio in **Co_2_W_11_** is 1:5.5, if the reaction was carried out in the stoichiometric ratio of starting metal salts (cobalt(II) acetate:sodium tungstate), a pink precipitate was isolated together with the **K-Co_2_W_11_** salt, which indicated that the Co(II) salt did not react completely. It must be mentioned that cobalt(II) chloride was also used as a cobalt source, with no significant differences in the reaction yield. Thus, acetate was chosen to decrease the number of different anions in the reaction medium. Modification of the W proportion to the 1:6 (Co:W) ratio reported in previous works [27] allowed us to reach almost quantitative reactions, showing reaction yields over 95% (based on W). As indicated in Table 2, further increase of the W ratio did not improve the final yield, which implies that the 1:6 ratio could be considered the ideal formulation for the sake of atomic efficiency of the reaction. 

**Figure 2:**
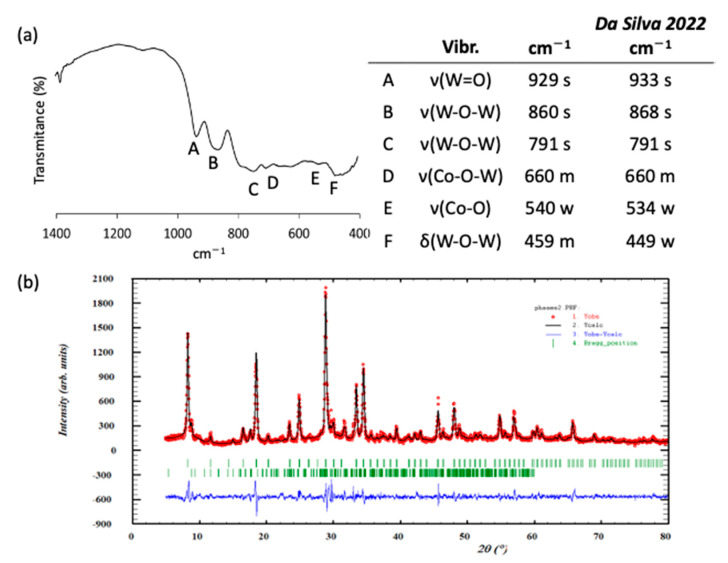
(**a**) FT-IR spectrum of **K-Co_2_W_11_** together with the assignment of the bands according to literature reports [34] (ν, stretching mode; δ, bending mode). (**b**) Fitting (black line) and experimental PXRD pattern (dotted curve) of **K-Co_2_W_11_**.

The purity and the nature of the highly hygroscopic **K-Co_2_W_11_** salt obtained as a green powder in a 96% yield using the 1:6 (Co:W) molar ratio of the starting reagents at pH = 7.5 was confirmed via PXRD, FT-IR spectroscopy (Figure 2), and XRF semiquantitative analysis, which revealed a 2:11 Co:W ratio for the final solid product (calculated for K_8_[Co_2_(H_2_O)W_11_O_39_]·17H_2_O: Co 3.46%, W 59.45%; Anal. Co 2.69%, W 57.45%). The vibrational analysis of the compound via FT-IR spectroscopy showed the most representative signals in the wavenumber range below 1000 cm^−1^. The assignment was performed based on previous results for these specific polyanions [34], which perfectly correlate with those reported in this work, as shown in Figure 2. The main absorption bands located at 929, 860, and 781 cm^−1^ can be assigned to vibrational modes ν_as_(W=O), ν_as_(W–O_b_–W), and ν_as_(W–O_c_–W), respectively, whereas the band at 660 cm^−1^ was assigned to the Co–O–W vibration of Co(II) ions in octahedral coordination mode. From the PXRD pattern, it can be concluded that the positions of the most characteristic diffraction maxima (2θ positions: 8.3, 18.6, 25.0, 28.9, 33.5, and 34.5) were in good agreement with those previously reported for this compound [34]. Additionally, the space group and lattice parameters of the crystalline compound were estimated via the fitting of the experimental pattern using Full Prof (version June 2022) software (Figure 2; see agreement factors in Table S1). This refinement afforded a cubic *Pm−3m* space group with a unit cell parameter of 10.71(2) Å for **K-Co_2_W_11_**. In addition, XPS analyses (Figure S2) were performed to prove the exclusive presence of Co(II) in the polyanion, as perceived from the positions of the Co 2p_3/2_ peak and its satellite at 781 eV and 786 eV, respectively, which perfectly correlates with those previously reported in the literature for Co(II) species [35]. 

#### 3.1.3. Reaction Time

Once the pH and the stoichiometry had been optimized, the mixture was heated to 100 °C over 20 min under reflux in order to avoid solvent losses. Different periods of reflux time were evaluated, ranging from 10 min to 1 h. When the Co^2+^ ion was incorporated within the POM framework, the color of the solution changed from pink to dark green, in good agreement with previously reported spectroscopic studies [36]. This fact facilitated the optimization of the reaction time via UV-Vis spectroscopy (Figure S3), revealing a change in the shape and the position of the maximum absorption peak from 500 to 600 nm. The results indicated that the reaction was completed after 20 min, because the spectrum of the reaction mixture perfectly coincided with that obtained for a freshly prepared solution of **K-Co_2_W_11_** in deionized water. This study allowed us to considerably shorten the long reaction times previously reported in the literature, with associated energy savings [29]. In the last precipitation step, a 1.5-fold stoichiometric excess of KCl proved to be enough for the efficient isolation of **K-Co_2_W_11_**, as indicated by the colorless nature of the resulting solution after the precipitation. 

#### 3.1.4. Transformation of **Co_2_W_11_** to **CoW_12_**

**Figure 3:**
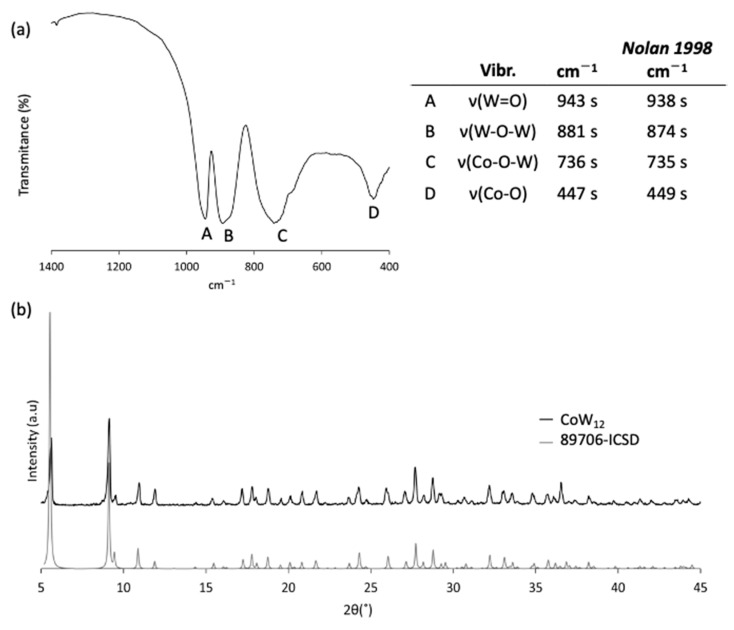
(**a**) FT-IR spectrum of **CoW_12_** together with the assignment of the bands according to literature reports [30] (ν, stretching mode). (**b**) PXRD diffraction pattern of **K-CoW_12_** in comparison to that simulated from single-crystal XRD data deposited in the ICSD database for K_5_H[CoW_12_O_40_]·15H_2_O (ICSD-89706) [28].

The last step of the reaction involves the structural rearrangement of the monosubstituted **Co_2_W_11_** anion to the plenary **CoW_12_** species in acidic media. It must be taken into account that the yield of this process can never reach 100% because the Co:W ratio is modified from 1:5.5 in **Co_2_W_11_** to 1:12 **CoW_12_** by the reassembly of the polyanion. This process starts with acidification to pH = 0 using either aqueous 1M HCl or 2M H_2_SO_4_, as previously reported in the literature [27,30]. When the acidic aqueous solution of the **K-Co_2_W_11_** salt was stirred under these conditions, a clear color change was perceived in the green dispersion, which indeed became a dark blue solution (Figure S4), indicating the transformation from **Co_2_W_11_** to **CoW_12_** and revealing the higher solubility of the latter. After complete dissolution in both acidic media, the mixture was left to evaporate at room temperature and the obtained products were significantly different. When using 1M HCl as a proton source, the formation of dark green needle-like crystals was observed after five days. To avoid the co-crystallization of secondary products with the desired **K-CoW_12_**, as well as tedious and lengthy recrystallization processes, the **K-Co_2_W_11_** and 1M HCl ratio was optimized to 1 g:10 mL. The nature and homogeneity of the product was confirmed using FT-IR spectroscopy and PXRD (Figure 3) as well as TGA analysis (Figure S5). The FT-IR spectrum compared well with those reported in the literature [30], showing the characteristic vibrational bands of the Keggin-type plenary anion in the region below 1000 cm^−1^. The main absorption bands located at 943, 881, and 736 cm^−1^ were assigned to the vibration modes ν_as_(W=O), ν_as_(W–O–W), and ν_as_(Co–O–W), respectively, whereas the band at 447 cm^−1^ was assigned to the Co–O vibration. The position and relative intensities of the diffraction maxima belonging to the experimental PXRD pattern perfectly matched those of the simulated patterns from single-crystal XRD data deposited in the ICSD database for K_5_H[CoW_12_O_40_]·15H_2_O (ICSD-89706) [28]. Conversely, the use of H_2_SO_4_ as a proton source afforded an additional white solid which was preliminarily identified as K_2_SO_4_ on the basis of FTIR spectroscopy (Figure S6); therefore, at least two recrystallization processes were needed to obtain a pure sample of **K-CoW_12_**. The consecutive recrystallization processes lowered the reaction yield considerably, such that the 88% yield observed for the reaction carried out with HCl dropped to 22% when H_2_SO_4_ was used.

**Figure 4:**
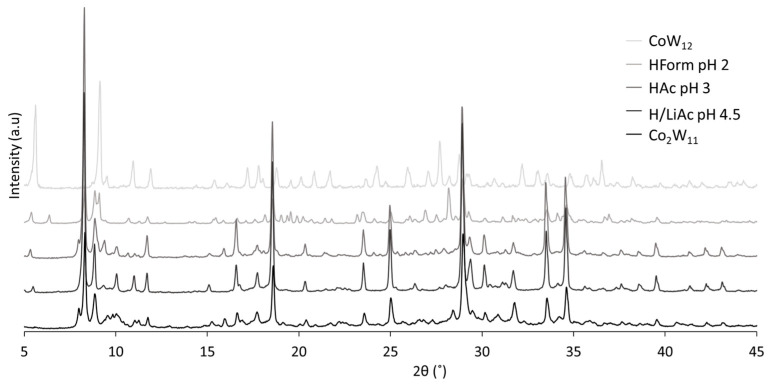
PXRD patterns for the solid compounds isolated after the acidification of **K-Co_2_W_11_** with aqueous 1M H/LiAc, 1M HAc, and 1M HForm compared to those recorded for **K-Co_2_W_11_** and **CoW_12_** synthesized using reported procedures.

To further understand the **Co_2_W_11_** to **CoW_12_** transformation through the acidic equilibria, the pH dependence of the procedure was studied in detail. For this purpose, solid **K-Co_2_W_11_** was suspended in diverse aqueous solutions of weak acids with different pH conditions: 1M formic acid (HForm, pH = 2), 1M acetic acid (HAc, pH = 3), and a 1M H/LiAc buffer (pH = 4.5) (Table S2). When the first drops of acid were added, it was observed that the color changed from green to dark blue and the precipitate began to dissolve. The solid dissolved completely when the pH of the solution was set at 2, and the solution displayed a deep blue color. The solids from the reactions carried out at pH values above 2 were filtered and the solutions were left to evaporate. The PXRD patterns acquired for the crystals formed in these solutions (Figure 4) were virtually identical to that displayed previously in Figure 2 for **K-Co_2_W_11_** (see Figure S7 for the FT-IR spectrum), with the main peak positions in 2θ (°) = 8.3, 18.6, 25.0, 28.9, 33.5, and 34.5, evidencing that no transformation took place in slightly acidic media. In contrast, the solid isolated from the reaction at pH = 2 corresponded to a mixture of **K-Co_2_W_11_** and **K-CoW_12_**, and diffraction maxima from both phases could be easily distinguished in the experimental PXRD pattern. Although the characteristic peaks of **K-CoW_12_** at 2θ (°) = 5.5, 9.0, 28.0, and 24.0 were present in the diffraction pattern, some other characteristic peaks from **K-Co_2_W_11_** were also found, such as those at 2θ (°) = 8.3, 18.6, 25.0, 28.9, 33.5, and 34.5. These observations indicate that a very acidic pH (<2) is required for the efficient transformation of **Co_2_W_11_** to **CoW_12_**. In conclusion, HCl remains the best option because it is associated with a reduction in time for the whole process from approximately a month to a week and, moreover, an increase in the total yield.

#### 3.1.5. Comparison with the Previously Reported Synthetic Methods

Table 3 displays a summary of the key parameters for the optimized synthetic procedure reported in this work in comparison to those found in the literature. Although the original synthesis has not been overly modified, the conditions selected in this work resulted in an increase of 20% in the total reaction, along with a significant decrease in the total reaction time from up to five weeks to only one week. In addition, some key points during the synthetic procedure were identified, such as the stoichiometry and the pH dependence of the rearrangement from **Co_2_W_11_** to **CoW_12_**. 

It must be mentioned that the one-pot synthesis was also considered a potentially interesting approach in order to save time and decrease the total cost of the reaction. Considering the acidic conditions shown to be required for **CoW_12_** formation, cobalt(II) acetate and sodium tungstate were dissolved in 1M HCl solution at a 1:12 stoichiometry ratio. However, the proposed reaction did not succeed. Only the pink precipitate that was identified as cobalt(II) salts of the [H_2_W_12_O_42_]^10−^ paratungstate anion (Figure S1) in Section 3.1.1 was obtained. Thus, it can be concluded that **CoW_12_** may not be directly accessed through a one-pot synthesis. 

#### 3.1.6. Electrochemical Characterization

**Figure 5:**
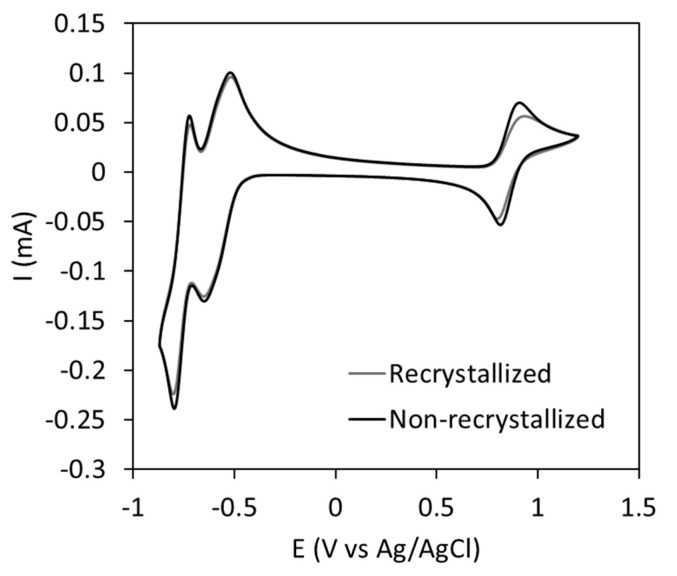
CV curves of **CoW_12_**-based electrolytes obtained from (i) solids isolated following previously reported methods, which include recrystallization processes; and (ii) the direct reaction reported in this work. Concentration **CoW_12_**: 5 mM, supporting electrolyte: 1M H/LiAc buffer. Second cycle represented at 50 mV s^−1^ scan rate.

The main purpose of this work was to optimize the synthetic procedure of POMs for use as electroactive species in RFBs. Thus, it was necessary to prove that the proposed procedure does not modify their electrochemical performance. Thus, the CV of **K-CoW_12_** isolated following the synthesis reported in this work is compared in Figure 5 with that of the salt prepared by strictly following previously published methods with the associated recrystallization steps [27]. Both samples exhibited virtually identical CV curves with two main regions separated by 1.7 V, which is a suitable voltage for RFBs. The region surrounding 0.9 V vs. Ag/AgCl is associated with a unique electron transfer from the Co oxidation process, while the second area from −0.5 to −0.9 V vs. Ag/AgCl corresponds to two consecutive two-fold electron transfers associated with W reduction. Both redox processes are highly reversible, as is evident from the 1:1 intensity ratio for the anodic and cathodic peaks associated with each redox process. The peak-to-peak separation is close to 59 mV in the case of the electron transference for Co and close to the half of this value for the double electron transferences for W, in good agreement with Nernst equation [37]. This electrochemical characterization reveals the suitability of the developed method for the incorporation of **CoW_12_** as electroactive species in RFBs. 

The electrochemical properties of the electrolyte revealed the feasibility of its implementation in a RFB. The high reversibility of the electron transferences ensures the long-term durability of the system. In addition, considering the displayed potential and the concentration of the POM, the expected energy density of the system was calculated to be 4.82 Wh L^−1^ in a symmetric cell configuration with the same electrolyte volume in both the anolyte and the catholyte. This value can increase considerably when taking advantage of the four electron transferences for W, reaching values of about 21.86 Wh L^−1^. This is indeed a competitive value, considering that the energy densities of commercial vanadium RFBs usually range from 20 to 33 Wh L^−1^ [38].

### 3.2. Development of the [Co(H_2_O)SiW_11_O_39_]^6−^ (**CoSiW_11_**) Electrolyte

**Figure 6:**
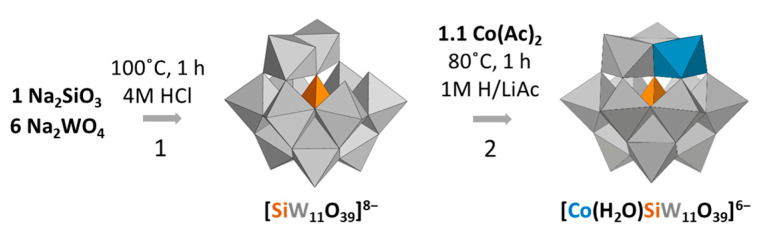
Scheme of the formation of the **CoSiW_11_** monosubstituted Keggin-type tungstosilicate. Synthesis of the **SiW_11_** monolacunary species (Reaction 1). Incorporation of the cobalt ion into the inorganic cluster skeleton (Reaction 2).

The monosubstituted species for Keggin-type POMs have been traditionally synthesized with little variation from the first reported synthetic procedure by Weakley and Malik in 1967 [39]. The reaction comprises two main steps (Figure 6): First, formation of the monolacunary K_8_[α-SiW_11_O_39_] (**K-SiW_11_**) precursor, in which one of the tungstate octahedral is removed in comparison to the plenary species [40], starting from the corresponding tungstate and silicate salts and setting the pH and temperature. Afterwards, the cobalt(II) ion is incorporated through the reaction between the metal salt and the monolacunary species in an aqueous (usually acetic acid/acetate buffers) medium to lead to monosubstituted [Co(H_2_O)SiW_11_O_39_]^6−^ (**CoSiW_11_**) anions. It is worth highlighting that the pure crystalline compound is obtained within less than one week with a high yield and without further purification steps. In fact, cobalt(II)-substituted POMs have been an object of study in diverse applications such as the electroreduction of CO_2_ to CO [41] and water oxidation electrocatalysis in acidic media [42]. In this context, provided that the **CoSiW_11_** synthesis is affordable in terms of time and yield, we tested whether the direct one-pot mixture of the metal sources in 1M H/LiAc supporting electrolyte could be suitable for the quantitative synthesis of **CoSiW_11_** [19] and whether the electrolyte displayed suitable physicochemical properties and electrochemical performance for implementation in RFBs. 

#### 3.2.1. One-Pot Preparation of **CoSiW_11_** Electrolyte

**Figure 7:**
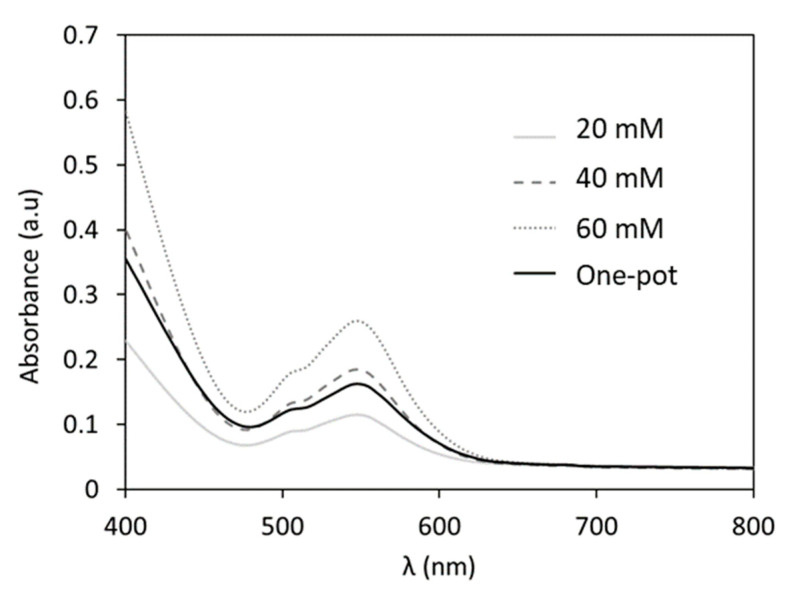
UV-Vis absorption spectra of the one-pot **CoSiW_11_** electrolyte together with those registered for three different concentrations of **K-CoSiW_11_** prepared following reported procedures and dissolved in the supporting electrolyte (1M H/LiAc).

The one-pot synthesis of **CoSiW_11_** implies the reaction of starting materials in a strict stoichiometric ratio (Co:Si:W 1:1:11; see Equation (2)) because the presence of additional species could interfere with the electrochemical performance of the electrolyte. Thus, all the reagents were dissolved in aqueous 1M H/LiAc buffer and left at reflux conditions for 12 h until the color of the solution changed from pale pink to dark red. This decreased the reaction time in comparison to the five-day traditional procedures and significantly simplified the preparation of the electrolyte. The quantitative formation of **CoSiW_11_** was confirmed via UV-Vis spectroscopy (Figure 7) using a calibration curve (Figure S8) with different concentrations of **K-CoSiW_11_** synthesized following the classical procedure. The absorbance of the band centered at 546 nm indicated that the reaction yield of the one-pot synthesis was as high as 78% by 12 h.

11 WO_4_^2−^ + SiO_3_^2−^ + Co^2+^ + 16H^+^ → [Co(H_2_O)SiW_11_O_39_]^6−^ + 7 H_2_O (2)

#### 3.2.2. Physicochemical Properties

Apart from the appropriate electrochemical performance, a suitable electrolyte for RFBs should display some specific physicochemical properties. The conductivity of the electrolyte must be high enough to avoid internal resistances in the electrochemical cell that could trigger possible energetic losses. Moreover, the concentration of the electrolyte must be precisely known, as the energy density of the system depends directly on it. Therefore, a detailed physicochemical characterization of the developed one-pot electrolyte was performed. The main characteristics are summarized in Table 4 in comparison to those obtained for a traditionally developed **CoSiW_11_**-based electrolyte from the crystalline compound. As shown in Table 4, the pH of both electrolytes was that of the buffered H/LiAc media used as a supporting electrolyte. Regarding the conductivity, higher conductivity values were observed for the one-pot electrolyte, which were associated with the remaining starting materials that had not reacted. However, if these salts show electrochemical activity, it is possible that they might negatively influence the final performance.

#### 3.2.3. Electrochemical Characterization

**Figure 8:**
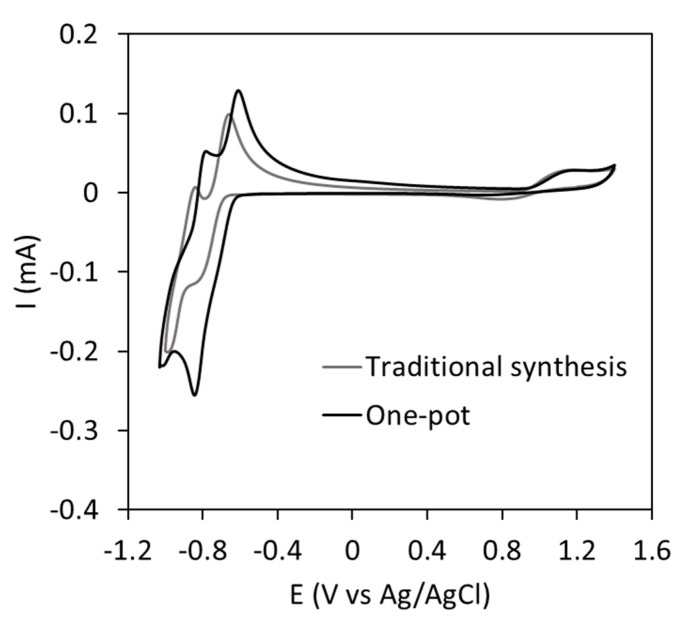
CV curves of the one-pot **CoSiW_11_** electrolyte and that of a solution of solid **K-CoSiW_11_**. Supporting electrolyte: 1M H/LiAc buffer. Curves correspond to the second cycle registered at 50 mV s^−1^ scan rate.

In order to prove that the proposed approach for the direct development of a POM-based electrolyte for RFBs is correct, the electrochemical activity of the electrolyte was studied via CV. Figure 8 shows the high similarity between the cyclic voltammogram registered for the one-pot electrolyte and that of a solution of solid **K-CoSiW_11_** [43] in aqueous 1M H/LiAc buffer. 

The CV showed two main regions separated by 1.9 V, which is a suitable voltage for RFBs and is even larger than that obtained for **CoW_12_**, which was ascribed to the changing position of the Co atom in the **CoSiW_11_** cluster. The CV revealed a unique electron transfer for the Co(II)→Co(III) oxidation at 1.05 V vs. Ag/AgCl and two consecutive two-fold redox reversible processes for W(VI)→W(V) at −0.73 V vs. Ag/AgCl and −0.88 V vs. Ag/AgCl, in good agreement with previously reported results [43]. This electrochemical characterization revealed the suitability of the developed method for the direct incorporation of the one-pot **CoSiW_11_** electrolyte into RFBs. Considering a symmetric system, the expected energy density is about 4.69 Wh L^−1^, whereas if the four electron transferences for W were used, values up to 24.44 Wh L^−1^ could be achieved. 

## 4. Conclusions

With the aim of creating an efficient POM-based RFB, the optimization of the synthesis of two electroactive species is reported in this work. The species are the cobalt(II)-containing Keggin-type plenary [CoW_12_O_40_]^6−^ (**CoW_12_**) and monosubstituted [Co(H_2_O)SiW_11_O_39_]^6−^ (**CoSiW_11_**) anions. During the optimization procedure, stoichiometry, starting materials, pH, and reaction time, among other parameters, were carefully evaluated in order to improve the yield and decrease the total cost of the reaction. 

The detailed study of the synthesis of K_4_H_2_[CoW_12_O_40_]·12H_2_O revealed critical steps of the reaction. First, specific pH conditions (7.5) and a 1:6 Co:W molar ratio of the starting reagents are crucial for the efficient isolation of the K_8_[Co_2_W_11_O_39_]·17H_2_O intermediate. Side products of this process were first identified using FT-IR, XRF, and PXRD and then avoided. Finally, the transformation to **CoW_12_** was found to be optimal when the reaction medium was acidified with aqueous HCl to pH = 0. These modifications involved an increase of up to 20% of the total reaction yield in comparison to reported procedures and the whole reaction time was reduced five-fold. It was also proven that an electrolyte based on **CoSiW_11_** cobalt(II)-monosubstituted tungstosilicate anions can be rapidly obtained by reacting the metal sources in a suitable supporting electrode such as the aqueous 1M H/LiAc buffer. The resulting one-pot electrolyte displayed suitable conductivities and redox properties, and the total cost and duration of the electrolyte production was decreased considerably. 

## Figures and Tables

**Table 1:** Reaction yields of the synthesis of **K-Co_2_W_11_** depending on pH of the initial solution.

pH	Yield K-Co_2_W_11_ (%)
6.5	46 ± 2
7	51 ± 1
7.5	96 ± 1
8	33 ± 2

Results are presented as the average of three consecutive repetitions for each pH value.

**Table 2:** Reaction yields of the synthesis of **K-Co_2_W_11_** depending on the molar ratio of the starting reagents.

Co(Ac)_2_:Na_2_WO_4_ Ratio	Yield K-Co_2_W_11_ (%)
1:5.5	89 ± 1
1:6	96 ± 1
1:6.5	91 ± 1

Results are presented as the average of three consecutive repetitions in each case.

**Table 3:** Timeline of the synthetic procedures reported in the literature for **CoW_12_** Keggin-type polyoxotungstate salts.

pH	Time	Precip. Agent	Co_2_W_11_ Yield	Acidification	Purification Method	CoW_12_ Yield	Reaction Time	Reference
6.5–7.5	10′	NH_4_(Ac)	85%	1M HCl	Recryst H_2_O ∙ 4	70%	Five weeks	[27]
6.5–7.5	10′	KCl	-	2M H_2_SO_4_	Recryst pH 4 H_2_O	47%	Two weeks	[30]
-	18–24 h	KCl	-	1M HCl	Recryst 0.1 M HAc	10%	Two weeks	[29]
7	20′	KAc	-	2M H_2_SO_4_	Ion exchange column	-	Undefined	[28]
7.5	20′	KAc	96%	1M HCl	-	88%	One week	This work

**Table 4:** Physicochemical properties of the one-pot **CoSiW_11_** electrolyte in comparison to those from a solution of **K-CoSiW_11_** prepared by dissolving a solid sample isolated following reported procedures in aqueous 1M H/LiAc buffer.

Properties	One-Pot	Traditional
pH	4.7	4.7
UV-Vis absorbance (nm)	546	546
Concentration (mM)	27	20
Conductivity (mS cm^−1^)	45.5	27.6

## Data Availability

Data is contained within the article or Supplementary Material.

## Supplementary Materials

The following supporting information can be downloaded at https://www.mdpi.com/article/10.3390/ma16145054/s1. Figure S1. FT-IR spectrum and assignment of the most significant bands [33] for the K_6_[{Co(H_2_O)_4_}_2_(H_2_W_12_O_42_)]·nH_2_O side product (ν, stretching mode and δ, bending modes). Table S1. Fitting parameters for the PXRD pattern of **K-Co_2_W_11_**. Figure S2. XPS spectrum of Co 2p peak of **K-Co_2_W_11_**. Figure S3. UV-Vis absorption spectra for the reaction mixture at t = 0 min and t = 20 min of the reaction, in comparison to a freshly prepared solution of **K-Co_2_W_11_** dissolved in deionized H_2_O. Figure S4. UV-Vis absorption spectra for **K-Co_2_W_11_** and **K-CoW_12_** in deionized H_2_O. Figure S5. TGA curve of **K-CoW_12_** under synthetic air conditions at 5 °C min^−1^. Figure S6. FT-IR spectrum for **K-CoW_12_** obtained after acidification of **K-Co_2_W_11_** in 2M H_2_SO_4_ with the corresponding band assignment for the **CoW_12_** and SO_4_^2−^ species, respectively [30,44]. Table S2. Yields of the reactions carried out under different pH conditions to evaluate the transition from **Co_2_W_11_** to **CoW_12_**. Figure S7. FT-IR spectra of the solid products isolated from the acidification of a solution of **K-CoW_12_** with aqueous HCl or H/LiAc. The band assignment revealed that the vibrational bands of the isolated from the reaction with HCl corresponded to **K-CoW_12_** (small letter) and that from the H/LiAc reaction corresponded to **K-Co_2_W_11_** (capital letter). Detailed band assignments for **K**-**Co_2_W_11_** and **K-CoW_12_** can be found in the manuscript in Figure 2 and Figure 3, respectively. Figure S8. UV-Vis absorption spectra (left) for different concentrations (20–120 mM) in 1M H/LiAc buffer of **K-CoSiW_11_** prepared following previously reported procedures, together with its calibration curve (right). References [30,33,44] are cited in the Supplementary Materials.
