# Supplementary material for: Systematic Approach to the Synthesis of Cobalt-Containing Polyoxometalates for Their Application as Energy Storage Materials
Source: Materials (Basel). 2023 Jul 17;16(14):5054. doi: 10.3390/ma16145054 (PMC10384885; doi:10.3390/ma16145054)
Supplement: Supplementary file 1 [file materials-16-05054-s001.zip › materials-2494322-supplementary.pdf]

Supporting Information

Systematic approach to the synthesis of cobalt-containing polyoxometalates for their application as energy storage materials

Ángela Barros [1*], Beñat Artetxe [2], Unai Eletxigerra [1], Estibaliz Aranzabe [1] and Juan M. Gutiérrez-Zorrilla [2]

[1] Surface Chemistry and Nanotechnologies Unit, Tekniker, Iñaki Goenaga 5, 20600 Eibar, Spain; unai.eletxigerra@tekniker.es (U.E.); estibaliz.aranzabe@tekniker.es (E.A.)
[2] Departamento de Química Orgánica e Inorgánica, Facultad de ciencia y Tecnología, Universidad del País Vasco UPV/EHU,, 48080 Bilbao, Spain; benat.artetxe@ehu.eus (B.A.), juanma.zorrilla@ehu.eus (J.M.G.-Z.)
* Correspondence: Correspondence: angela.barros@tekniker.es

Table of contents

Figure S1. FT-IR spectrum and assignation of the most significant bands [33] for the $K_6[\{Co(H_2O)_4\}_2(H_2W_{12}O_{42})]\cdot nH_2O$ side product (ν, stretching mode and δ, bending modes). 1

Table S1. Fitting parameters for the PXRD pattern of **K-Co$_2$W$_{11}$**. 1

Figure S3. UV-Vis absorption spectra for the reaction mixture at t = 0 min and t = 20 min, in comparison to the freshly prepared solution of **K-Co$_2$W$_{11}$** dissolved in deionised H_2O. 2

Figure S4. UV-Vis absorption spectra for **K-Co$_2$W$_{11}$** and **K-CoW$_{12}$** in deionised H_2O........ 2

Figure S5. TGA curve of **K-CoW$_{12}$**. 3

Figure S6. FT-IR spectrum of **K-CoW$_{12}$** obtained after acidification of **K-Co$_2$W$_{11}$** in 2M H_2SO_4 with the corresponding assignment of the bands for **CoW$_{12}$** and SO_4^{2-} species, respectively [30], [44]. . 3

Table S2. Yields of the reactions carried out at different pH conditions to evaluate the transition from **Co$_2$W$_{11}$** to **CoW$_{12}$.**........ 3

Figure S7. FT-IR spectra of the solid products isolated from the acidification of a solution of K-CoW$_{12}$ with aqueous 1M HCl or H/LiAc. The assignment revealed that the solid isolated from the reaction with HCl corresponds to **K-CoW$_{12}$** (small letters), whereas that from H/LiAc agrees with **K-Co$_2$W$_{11}$** (capital letter). Find the detailed band assignment for **K-Co$_2$W$_{11}$** and **K-CoW$_{12}$** in the manuscript enclosed in Figures 2 and 3, respectively. 4

Figure S8. UV-Vis absorption spectra (left) for different concentrations (20-120 mM) in 1M H/LiAc buffer of **K-CoSiW$_{11}$** prepared following previously reported procedures, together with its calibration curve (right). 4

References 4

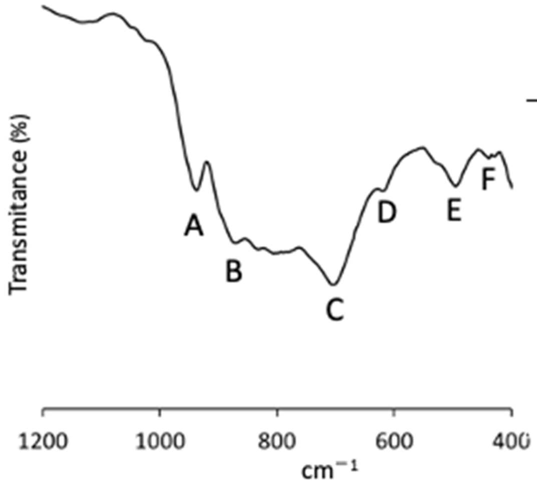

	Vibr.	cm⁻¹	Gimenez-Saiz 1995 cm⁻¹
A	ν(W=O)	936 s	937 s
B	ν(W-O-W)	879 m	876 m
C	ν(Co-O)	700 s	699 m
D	ν(Co-O-W)	524 w	527 w
E	ν(Co-O)	493 m	495 m
F	δ(W-O-W)	427 w	422 w

Figure S1. FT-IR spectrum and assignation of the most significant bands [33] for the $K_6[\{Co(H_2O)_4\}_2(H_2W_{12}O_{42})]\cdot nH_2O$ side product (ν, stretching mode and δ, bending modes).

Table S1. Fitting parameters for the PXRD pattern of **K-Co₂W₁₁**.

Lattice parameters	Agreement factors
Cubic, *Pm*-3*m*	R_{Bragg} = 2.59
a = 10.71(2) Å	χ^2 = 2.16

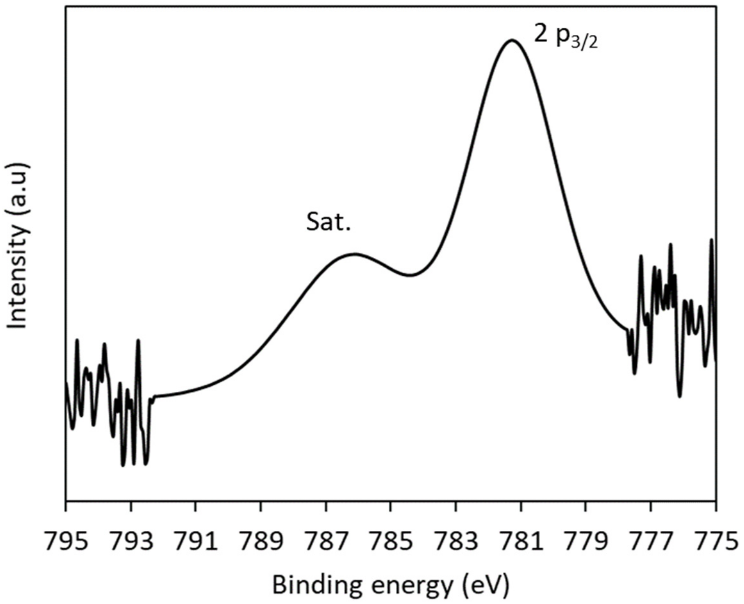

Figure S2. XPS spectrum of Co 2p peak for **K-Co₂W₁₁**.

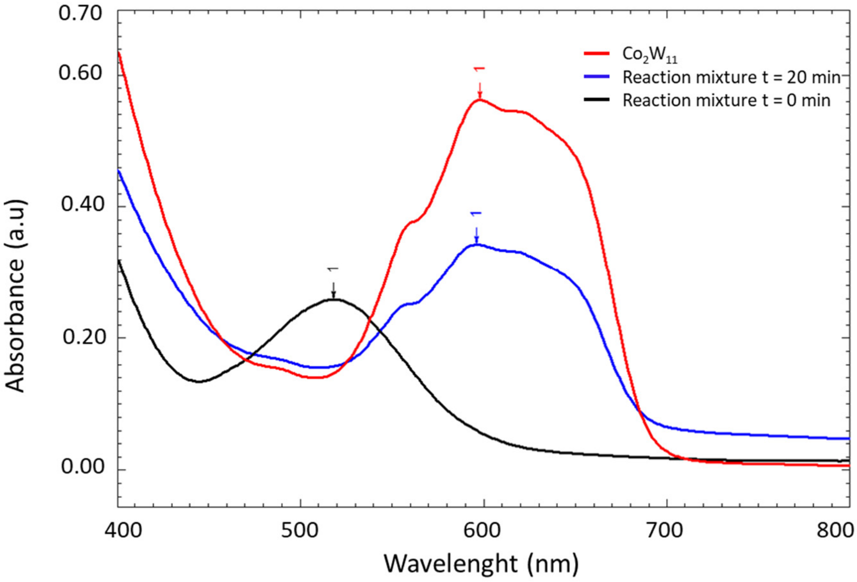

Figure S3. UV-Vis absorption spectra for the reaction mixture at t = 0 min and t = 20 min, in comparison to the freshly prepared solution of **K-Co_2W_{11}** dissolved in deionised H_2O.

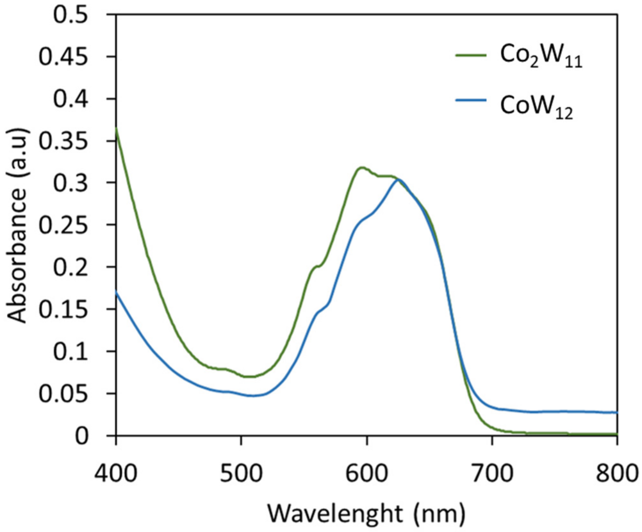

Figure S4. UV-Vis absorption spectra for **K-Co_2W_{11}** and **K-CoW_{12}** in deionised H_2O.

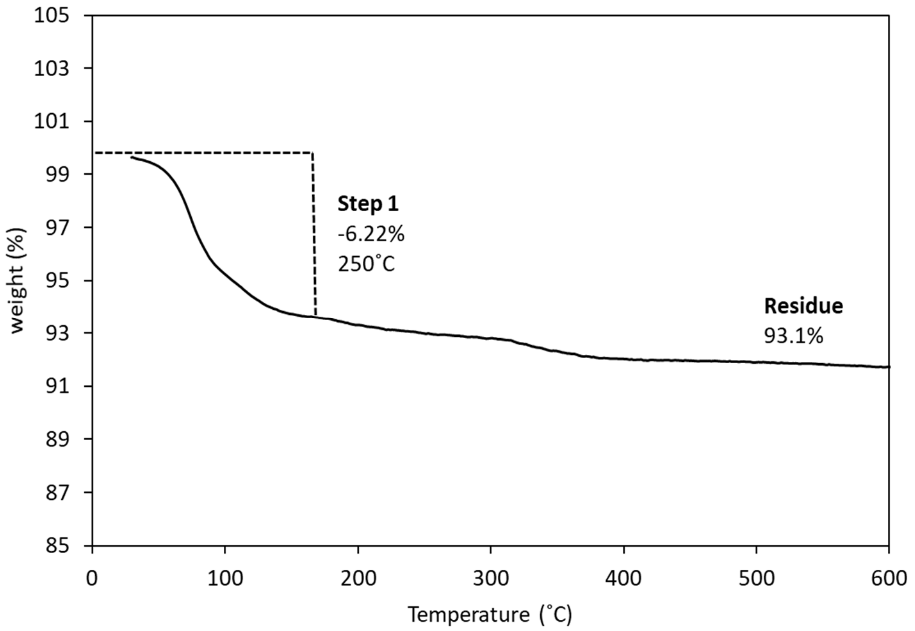

Figure S5. TGA curve of **K-CoW$_{12}$**.

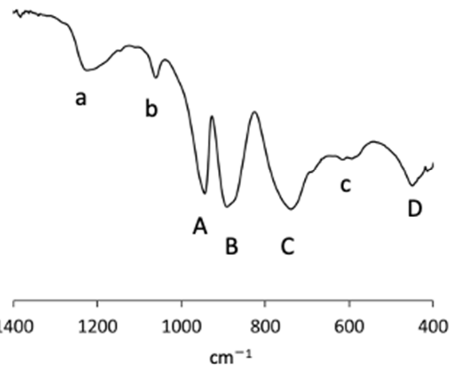

	Vibr.	cm⁻¹	*Nolan 1998* cm⁻¹	*Pretsch 2020* cm⁻¹
a	S=O	1223 m		1225
b	S-O	1059 m		1060
A	ν(W=O)	943 s	938 s	
B	ν(W-O-W)	881 s	874 s	
C	ν(Co-O-W)	736 s	735 s	
c	S-O	615 w		612
D	ν(Co-O)	447 s	449 s	

Figure S6. FT-IR spectrum of **K-CoW$_{12}$** obtained after acidification of **K-Co$_2$W$_{11}$** in 2M H_2SO_4 with the corresponding assignment of the bands for **CoW$_{12}$** and SO_4^{2-} species, respectively [30], [44].

Table S2. Yields of the reactions carried out at different pH conditions to evaluate the transition from **Co$_2$W$_{11}$** to **CoW$_{12}$.**

Acid	pH	Yield (%)
**1M HCl**	0	88±10
**1M H_2SO_4**	0	22±7
**1M HCOOH (HForm)**	2	-
**1M CH_3COOH (HAc)**	3	-
**1M CH_3COOLi/CH_3COOH (H/LiAc)**	4.5	-

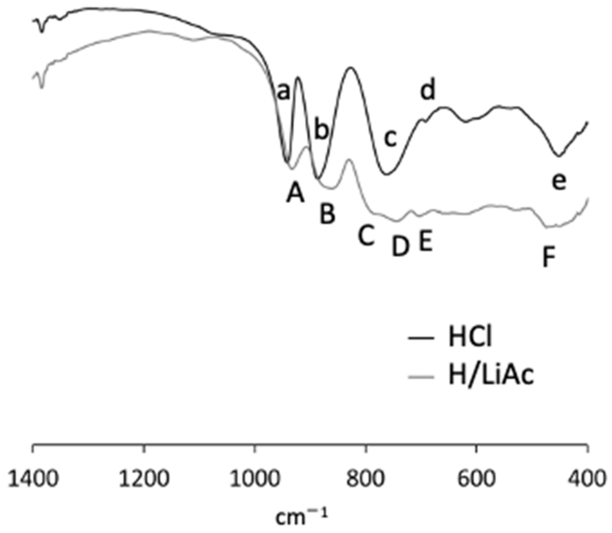

Figure S7. FT-IR spectra of the solid products isolated from the acidification of a solution of **K-CoW**$_{12}$ with aqueous 1M HCl or H/LiAc. The assignment revealed that the solid isolated from the reaction with HCl corresponds to **K-CoW**$_{12}$ (small letters), whereas that from H/LiAc agrees with **K-Co**$_{2}$**W**$_{11}$ (capital letter). Find the detailed band assignment fo**r K-Co**$_{2}$**W**$_{11}$ and **K-CoW**$_{12}$ in the manuscript enclosed in Figures 2 and 3, respectively.

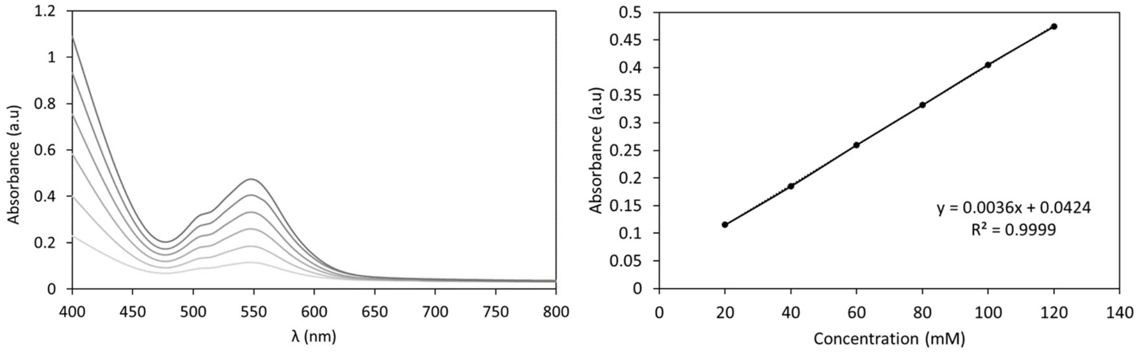

Figure S8. UV-Vis absorption spectra (left) for different concentrations (20-120 mM) in 1M H/LiAc buffer of **K-CoSiW**$_{11}$ prepared following previously reported procedures, together with its calibration curve (right).

References

30. Nolan, A.L.; Burns, R.C.; Lawrance, G.A. Oxidation of $[Co^{II}W_{12}O_{40}]^{6-}$ to $[Co^{III}W_{12}O_{40}]^{5-}$ by Peroxomonosulfate in Strong and Weak Acid Solutions, an Example of Zero-Order Kinetics. *J. Chem. Soc. Dalton Trans.* **1998**, 3041–3048, doi:10.1039/a804598d.
33. Gimenez-Saiz, C.; Galan-Mascaros, J.R.; Triki, S.; Coronado, E.; Ouahab, L. $[(Co(H_2O)_4)_2(H_2W_{12}O_{42})]_N^{6n-}$: A Novel Chainlike Heteropolyanion Formed by Paradodecatungstate and Cobalt(II) Ions. *Inorg. Chem.* **1995**, *34*, 524–526, doi:10.1021/ic00106a015.
44. Pretsch, E.; Bühlmann, P.; Badertscher, M. *Structure Determination of Organic Compounds: Tables of Spectral Data*; Springer Berlin Heidelberg: Berlin, Heidelberg, 2020; ISBN 978-3-662-62438-8.
